# Sexual Assault and the Association With Health, Quality of Life, and Self-Efficacy in the General Norwegian Population

**DOI:** 10.1177/0886260520926307

**Published:** 2020-06-09

**Authors:** Inger Schou-Bredal, Tore Bonsaksen, Øivind Ekeberg, Laila Skogstad, Tine K. Grimholt, Anners Lerdal, Trond Heir

**Affiliations:** 1 University of Oslo, Norway; 2 Oslo University Hospital, Norway; 3 Oslo Metropolitan University, Akershus, Norway; 4 VID Specialized University, Sandnes, Norway; 5 Lovisenberg Diakonale Hospital, Oslo, Norway; 6 Norwegian Centre for Violence and Traumatic Stress Studies, Oslo, Norway

**Keywords:** health, sexual assault, general self-efficacy, posttraumatic stress disorder, quality of life

## Abstract

The lifetime prevalence of sexual assault was examined in a representative sample of the general Norwegian adult population (*n* = 1,792), in addition to the association between sexual assault and health, quality of life, and general self-efficacy. Respondents completed questionnaires assessing these factors. Overall, 6.7% (*n* = 120) of the respondents (10.9% of women and 1.9% of men) reported an experience of sexual assault. Respondents in the sexual assault group reported significantly worse mental and physical health as well as poorer quality of life and lower self-efficacy, compared with those without sexual assault experience. The most prevalent mental problems in the sexual assault group were depression (61.7%), sleep problems (58.3%), eating disorders (26.7%), and posttraumatic stress disorder symptoms at a clinical level (25.0%). The most prevalent physical problems were chronic pain (47.5%) and musculoskeletal disease (30.8%). The proportions of physical and mental health problems were not significantly different between male and female victims. Results indicated that having experienced sexual assault during one’s life appears to be associated with lifetime occurrence of multiple health problems for both genders and reduces a person’s perceived general self-efficacy and quality of life.

## Introduction

Sexual assault (SA) is a common form of trauma worldwide and represents a serious public health problem: 18% to 51% of women report that they have been sexually assaulted during their lifetime (Black et al., 2011; Elliott et al., 2004; Masho et al., 2005), compared with 1% to 9% of men (Basile et al., 2015; Black et al., 2011; Breiding et al., 2014; Elliott et al., 2004). Women are more likely to experience most types of interpersonal violence than men, including sexual abuse in childhood (Tjaden & Thoennes, 1998). In contrast to the broader literature on trauma, which generally assesses lifetime experiences of different kinds of trauma (including SA), the literature on SA has mostly focused on incidents in childhood or adolescence, and in female adults (Dworkin et al., 2017). Despite the increased attention on SA, research on male victims remains limited. Thus, research regarding SA in men has often been restricted to all-male environments (Peterson et al., 2011). Only a few studies have evaluated SAs in male participants compared with female participants considering their characteristics and the impact of SA in the general population (Breiding et al., 2014; Elliott et al., 2004).

SA has consequences for the victim, as well as costs for society. Consequences for the victim may include immediate physical harm and increased risk of sexually transmitted illnesses, pregnancy, mental health problems, and chronic physical health problems (Ciccone et al., 2005; Fanslow & Robinson, 2004). There is an immediate medical cost for those who seek help. About one third of rape victims seek mental health services (Young et al., 2018). SA is also associated with decreased employment (Haagsma et al., 2012; Livingston, 2009), and some rape victims might be eligible to receive disability benefits. However, most cases of SA are not reported to the police and most victims do not seek health services, making effective strategies for detection, prevention, and treatment difficult (Feldhaus et al., 2000). According to previous research, female victims are more likely than male victims to be injured during a SA, to use medical services, and to report the assault to the police (Kimerling et al., 2002). However, underreporting is more likely among male than female victims. Male victims of SA also take significantly longer to disclose or seek medical and/or mental health services, if they do so at all (Tjaden & Thoennes, 2006).

Over time, people who have experienced SA are more likely to experience chronic medical conditions, such as fibromyalgia, gastrointestinal (GI) symptoms, infertility, chronic fatigue, and chronic pain (Ciccone et al., 2005; Saps et al., 2009; ter Wolbeek et al., 2006). Acute psychological distress in the aftermath of SA is common. The odds of receiving a posttraumatic stress disorder (PTSD) diagnosis in the year following the assault are more than 6 times higher than among persons in the general population (Kilpatrick et al., 1992). The lifetime prevalence of PTSD associated with SA has been found to range from 26.6% to 45.2% (Walsh et al., 2012). In Norway, a recent population study found that the prevalence of current PTSD was 3.8 % for men and 8.5% for women, with SA being one of the most common events causing it (Heir et al., 2019). The Norwegian point prevalence rates appear to be higher than lifetime prevalence rates found in many other countries (Duckers et al., 2016). This finding points toward the “vulnerability paradox,” indicating that those living in countries with more resources (such as Norway) have higher, rather than lower, PTSD risk (Heir et al., 2019). Augmenting this line of reasoning concerned with cross-national diversity in health outcomes following SA, health differences in Norway between persons with and without lifetime SA may be notable.

Furthermore, the quality of social, family, and intimate-partner relationships often decline following a SA, and there is an increased risk of sexual dysfunction (Dennerstein et al., 2004). Survivors of SA are 6 times more likely to attempt suicide than in the general population (Tomasula et al., 2012). A review of mental disorders in female survivors of adult SA found that 13% to 51% met the diagnostic criteria for depression, 12% to 40% experienced anxiety, and 13% to 49% had developed alcohol abuse disorder (Campbell et al., 2009) and perceived their health status as poor (Masho et al., 2005). The few studies that have investigated self-efficacy in relation to SA found that higher perceived self-efficacy was associated with lower rates of depression and PTSD after SAs in female victims (Cieslak et al., 2008; Regehr et al., 1999). Several studies have found that female survivors of SA are more likely to have low self-efficacy (Cieslak et al., 2008; Regehr et al., 1999; Voller et al., 2015). We identified only one study that investigated self-efficacy and SA in male victims (Voller et al., 2015). Among male veterans, lower self-efficacy was associated with more severe psychiatric symptoms.

Most studies of SA are based on convenience samples, such as persons who report to the police or who seek mental health treatment. However, these convenience samples cannot be used to provide prevalence estimates of SA in the general population, as most instances of SA are not reported. Population-based studies are important to assess the consequence of SA on the mental and physical health of both genders. Most of the studies mentioned above only included female victims or focused on childhood SA. To our knowledge, no previous study has investigated SA in the general population including both genders and compared victims of SA with non-victims with regard to general self-efficacy (GSE) and mental health, physical health, and quality of life (QoL). Generally, it is believed that men are less negatively affected by SA. However, there is some evidence that SA is as psychologically distressing to male victims as it is to female victims and might even be associated with poorer outcomes (Peterson et al., 2011). Therefore, including male victims in research is important to determine whether factors associated with SA vary by gender. To date, knowledge about male victims of SA is still less than that for women.

The study was conducted to answer the following research questions:

**Research Question 1:** What is the prevalence of lifetime SA in men and women in the general Norwegian population?
**Research Question 2:** Do persons with lifetime SA report poorer health, QoL, and GSE, compared with persons without lifetime SA?

## Method

### Study Design

The Norwegian Population Study is a cross-sectional survey. Representativeness was ascertained by comparing selected demographic characteristics of the sample data with data from Statistic Norway, 2015. The collected data reflect a variety of health conditions in the general population and will provide national normative scores related to several questionnaires used for assessing symptoms, attitudes, and behavior. No identifying information was collected. Those who provided informed consent to participate completed the questionnaires and returned them in a sealed envelope.

### Participants and Procedures

Participants were recruited through a postal mailing sent to a random sample of 5,500 adults (≥18 years of age) selected by the National Population Register and stratified by age, gender, and geographic region. Of these participants, nine persons had died, 21 were unable to fill out the questionnaire because of comorbidity or old age, and 499 envelopes had invalid addresses. Thus, 4,971 were eligible and 1,792 persons (response rate 36%) completed the questionnaires and returned them to the researchers. The data collection took place in 2014 and 2015. The postal survey was carried out anonymously and at request, the Regional Committee for Medical and Health Research Ethics required no further formal ethical approval.

### Definitions of SA

Definitions of SA vary considerably across research samples. Here, SA refers to attacks such as rape or attempted rape and sexual acts through force or threat of harm.

### Measures

Lifetime experience of SA was measured with the Life Event Checklist for *Diagnostic and Statistical Manual of Mental Disorders* (5th ed.; *DSM-5*; American Psychiatric Association [APA], 2013) (LEC-5; Gray et al., 2004). The questionnaire has demonstrated adequate test–retest reliability for the scale items (mean κ = 0.61) and moderate correlation (*r* = –.55, *p* < .001) with trauma-related mental disorders measured with the Traumatic Life Events Questionnaire (Gray et al., 2004). In the list of life events, participants were asked whether they had experienced SA (being subjected to rape or attempted rape, or made to perform any type of sexual act through force or threat of harm). They were given six options: *it happened to me*; *I witnessed it*; *it happened to a person close to me*; *it occurred as part of my work*; *not sure*; and *does not apply to me*. Participants were classified as being a victim of lifetime SA if they responded that this had happened to them. They were also asked, “How many years ago is it since it happened?” and “Did you perceive the SA as a threat to your life or cause of serious injury?” On a scale from 0 (*no influence*) to 10 (*extreme influence*), they were asked to rate the impact that the incident had had on their functioning/their life.

### Mental Health Problems

In this study, we asked, “Below you will find a list of mental health problems. Do you have, or have you had, any of these problems?” This list contained anxiety, depression, sleep problems, attempted suicide, self-injury, and eating disorders. Participants were given the options: *no*; *yes earlier, but not in the last month*; and *yes, this month*. Participants who answered with *yes earlier*, or *yes, this month* were classified as cases. They were also asked if they consumed alcohol. Response options were *no*; *sometimes*; *every week*; *daily*; or *several times a day*. Respondents, who answered *no* or *sometimes* were classified as not consuming alcohol.

### PTSD

The checklist for *DSM-5* (PCL-5) was used to measure PTSD symptoms. The PCL-5 is a 20-item self-administered questionnaire that assesses the full domain of the *DSM-5* PTSD diagnosis (Weathers et al., 2013). It consists of four subscales, corresponding to each of the symptom clusters in the *DSM-5*. The symptoms endorsed were specifically linked to the index event identified in the PCL-5. Each item was scored on a 5-point Likert-type scale (0, *not at all*; 1, *a little*; 2, *moderately*; 3, *quite a bit*; and 4, *extremely*) in rating to which extent participants were bothered by the 20 symptoms over the past month. The *DSM-5* diagnostic guidelines of the APA (2013) were applied to the PCL-5 to categorize participants as fulfilling the PTSD symptom criteria or not. Participants indicating scores of 2 or above on at least one of five re-experiencing symptoms, one of two avoidance symptoms, two of seven symptoms of negative alterations in cognition and mood, and two of six arousal symptoms were classified as fulfilling the PTSD symptom criteria (Ashbaugh et al., 2016; Weathers et al., 2013).

The Norwegian version of the PCL-5 was developed through an alternating procedure of translations and back-translations (Vijver & Hambleton, 1996). The original authors approved the final English back-translation. The PCL-5 has been shown to have good internal consistency (Cronbach’s α = .95 for the total scale, α > .79 for each of the four subscales), strong convergent validity (*r* = .81 with Impact of Events Scale–Revised [IES-R] scale score), and good divergent validity (*r* = .61 with Center for Epidemiological Studies–Depression Scale; significantly lower than the correlation with IES-R) (Ashbaugh et al., 2016).

### Physical Health Problems

In addition, we asked, “Below you will find a list of physical health problems. Do you have, or have you had, any of these problems?” Listed physical health problems were *GI disease, fibromyalgia*, *chronic musculoskeletal disease*, and *chronic pain*. For all of the listed mental and physical health problems, the response alternatives were *no*; *yes previously, but not during the last month*; and *yes, during the last month*. Participants who answered *yes previously* or *yes, during the last month* were classified as cases.

### Perceived QoL

The participants were asked to indicate on a scale from 0 (*extremely poor*) to 10 (*excellent*) how they would rate their perceived QoL and health in the last week. These items are based on two items of the European Organization for Research and Treatment of Cancer (EORTC) questionnaire (Aaronson et al., 1993). According to EORTC, these two items combined are defined as global health status/QoL. The two items measuring health and QoL, respectively, correlated strongly (*r* = .77, *p* < .001; Bonsaksen, Ekeberg, et al., 2019). Then, the global health/QoL measure was established by calculating the average score for the two items and by transforming this score to represent a point on a 0 to 100 scale (i.e., multiplying the raw score by 10). A high score for the global health status/QoL represents high QoL.

### GSE

The *General Self-Efficacy Scale* (Schwarzer & Jerusalem, 1995) measures self-beliefs related to coping with the demands, tasks, and challenges of life in general. Respondents rate the 10 GSE statements from 1 (*not at all true*) to 4 (*exactly true*). Examples of statements are *I can always manage to solve difficult problems if I try hard enough* and I *am certain that I can accomplish my goals*. The GSE score is calculated as the sum of all item scores, the sum score ranging between 10 and 40, with higher scores indicating higher GSE. Factor analysis of the GSE has consistently produced a one-factor solution, which was confirmed in a previous study using the same general population sample as in the current study (Bonsaksen, Lerdal, et al., 2019). For both studies, thus, Cronbach’s α was .92.

### Statistical Analyses

All analyses were implemented after examination of the data. We determined our sample size, all data exclusions (if any), all manipulations, and all measures in the study, from the 21 Word Solution (Simmons et al., 2012). Our sample size calculation, performed before the commencement of the study, was based on the assumptions that we wish to be able to document (a) differences in group percentages as small as six percentage points, (b) between two (identically sized) groups, (c) percentages around 50 (e.g., 50% vs. 44%), (d) with a maximum risk of 5% of committing a type 1 error, (e) with a statistical power of 80%, and (f) an expected response rate of 40%. Thus, based on the sample size calculations, we needed to send our questionnaire to 5,406 persons: [(1.96 + 0.84) × ((50 × (100 – 50)) + (44 × (100 – 44))]/(50 – 44) × (50 – 44) × (100/40) × 2. Subsequently, the questionnaire was sent to 5,500 persons.

Data were analyzed using IBM SPSS Statistics for Windows (IBM Corporation, 2019). General descriptive analyses were conducted as appropriate. Data are presented as numbers with percentages or means with the 95% confidence interval (CI). When comparing dichotomous/nominal variables between two groups, chi-square tests were used. Independent-samples *t* tests were used for comparing continuous variables between the two groups, and Cohen’s *d* was used as effect size (Cohen, 1992). Pearson’s correlation coefficient was calculated to analyze any association between two continuous variables. Risk estimates were calculated using 2 × 2 tables. The significance level *p* was set at 5%.

## Results

The sociodemographic characteristics of the respondents with (SA group) and without (non-SA group) experience of SA during their lifetime (in the total sample and according to gender) are presented in Table 1.

**Table 1.** Sociodemographic Characteristics of Respondents With and Without Experience of SA by Gender.

	Participants With SA^a^			Participants Without SA^b^			*p* ^c^
	Total	Female^d^	Male^d^	Total	Female^d^	Men^d^	
	(*n* = 119)*n* (%)	(*n* = 103)*n* (%)	(*n* = 16)*n* (%)	(*n* = 1,664)*n* (%)	(*n* = 844)*n* (%)	(*n* = 820)*n* (%)	
Age (years)^e^							.06
18–35	26 (21.8)	25 (24.3)	1 (6.3)	282 (16.9)	170 (20.1)	112 (13.7)	
36–50	37 (31.1)	34 (33.0)	3 (18.8)	420 (25.2)	239 (28.5)	181 (22.1)	
51–65	38 (31.9)	31 (30.1)	7 (43.8)	515 (30.9)	236 (28.0)	279 (34.0)	
≥66	18 (15.1)	13 (12.6)	5 (31.3)	447 (26.9)	199 (23.6)	248 (30.2)	
Resident in a							.21
Village (<2,000 inhabitants)	19 (16.0)	15 (14.5)	4 (25.0)	339 (20.4)	165 (19.5)	174 (21.5)	
Town (2,000–19,999)	44 (37.0)	41 (39.8)	3 (18.8)	441 (26.5)	223 (26.4)	218 (26.6)	
Small city (20,000–99,999)	24 (20.2)	19 (18.4)	5 (31.3)	402 (24.2)	213 (25.2)	189 (23.0)	
Large city (≥100,000)	31 (26.1)	28 (27.2)	3 (18.8)	463 (27.8)	234 (27.7)	229 (27.9)	
Marital status							<.01
Married/cohabiting	71 (59.7)	58 (56.3)	13 (81.3)	1,242 (74.6)	621 (54.6)	621 (52.1)	
Single	21 (17.6)	21 (20.4)	0	208 (12.5)	112 (13.3)	96 (11.8)	
Divorced/separated	11 (9.2)	11 (10.7)	0	86 (5.2)	48 (5.7)	38 (4.7)	
Widowed	5 (4.2)	3 (2.9)	2 (12.5)	69 (4.1)	46 (5.5)	23 (2.8)	
Boy/girlfriend	11 (9.2)	10 (9.7)	1 (6.3)	78 (4.7)	41 (4.9)	37 (4.5)	
Education							.58
≥12 years	67 (56.3)	59 (57.3)	8 (50.0)	882 (53.0)	458 (54.6)	424 (52.1)	
Work status							<.001
Employed	70 (58.8)	63 (61.2)	7 (43.8)	1,031 (62.0)	541 (64.1)	490 (59.8)	
Undergoing education	9 (7.6)	8 (7.8)	1 (6.3)	81 (4.9)	47 (5.6)	34 (4.1)	
Unemployed	2 (1.7)	2 (1.9)	0	21 (1.3)	9 (1.1)	12 (1.5)	
Retired	20 (16.8)	13 (12.6)	7 (43.8)	440 (26.4)	203 (24.1)	237 (28.9)	
On disability benefits	18 (15.1)	17 (16.5)	1 (6.3)	91 (5.5)	44 (5.2)	47 (5.7)	

*Note.* SA = sexual assault.

a In the SA group, one person did not provide age and one did not provide gender, and these were excluded from the analysis.

b In the non-SA group, 19 persons did not provide age and eight did not provide gender, and these were excluded from the analysis.

c Statistical tests (chi-square) are comparisons of participants with SA versus without SA, with men and women together.

d In addition to the data displayed in Table 1, gender proportions were significantly different between participants with SA (13.4% males) and participants without SA (49.3% males, *p* < .001).

e Age when answering the questionnaires.

### Prevalence

There were 120 individuals (6.7%) who reported that they had experienced SA during their lifetime (10.9% among women and 1.9% among men). Of these, 103 (85.8%) were women and 16 (13.3%) were men. The prevalence of reported SA was not significantly different according to age group. Among the respondents who received disability benefits, 17.1% reported that they had experienced SA during their lifetime versus 6.2% among those that did not receive disability benefits (Table 1).

On average, the SA had occurred 24.7 years ago (range: 2–68). Eleven of the 16 men (69%) experienced SA as a child or adolescent (at or before the age of 15 years), compared with 30 out of 103 (29%) among women. The majority (*n* = 83, 69%) experienced SA before they were 30 years of age. One man (6%) experienced SA when he was above 50 years of age, compared with six women (6%). A total of 73 (60.8%) reported that they perceived the SA as a threat to their life or that the incident had caused serious injury. Of these, four (25%) were men and 69 (66.9%) were women. The SA group scored a mean of 6.5 (95% CI [6.0, 7.0]) regarding the impact of the SA on their functioning/life. There was no significant difference (*p* = .06) between female and male victims regarding the impact of SA on their functioning life; that is, men scored 4.9 (95% CI [3.0, 6.8]) and women scored 6.8 (95% CI [6.3, 7.3]).

### Alcohol Consumption

There was no significant difference between the SA and non-SA groups regarding daily consumption of alcohol (2.5% vs. 2.2%, respectively). None of the 16 men in the SA group reported that they had daily consumption of alcohol, while 2.9% of the women did.

### Mental and Physical Health

There was a significant difference in reported mental and physical health between the two groups (Table 2). Thirty respondents (25%) in the SA group fulfilled the PTSD symptom criteria compared with 112 respondents (6.7%) in the non-SA group. There was no significant difference between genders regarding PTSD in the SA group, but there was a significant difference between genders regarding PTSD in the non-SA group (Table 2). In the SA group, 19 (15.8%) individuals had attempted suicide, 19 (15.8%) had performed self-injury, and 32 (26.7%) had or had experienced an eating disorder. There was no significant difference between the genders regarding reported mental health in the SA group. In the non-SA group, women reported significantly more mental health problems than men (16.4% vs. 11.8%, respectively, *p* < .01). The SA group also reported significantly more physical health problems than the non-SA group (29.4% vs. 12.9%, respectively, *p* < .01). In the SA group, there was no significant difference between men and women regarding reported physical health problems.

**Table 2.** Lifetime Prevalence of Mental and Physical Health Problems Reported by Participants With and Without Experience of SA, and Risk Estimates for the SA Group.

	Participants With SA^a^			Participants Without SA^a^			*p* *	Risk estimate
	Total (*n* = 120)	Women (*n* = 103)	Men (*n* = 16)	Total (*n* = 1,664)	Women (*n* = 844)	Men (*n* = 820)		
	*n* (%)	*n* (%)	*n* (%)	*n* (%)	*n* (%)	*n* (%)		
Mental health								
Anxiety	57 (47.5)	51 (49.5)	6 (37.5)	321 (19.2)	187 (22.2)	133 (16.2)	<.001	2.5
Depression	74 (61.7)	66 (64.1)	8 (50.0)	444 (26.6)	252 (29.9)	192 (23.4)	<.001	2.4
PTSD	30 (25.0)	28 (27.2)	2 (12.5)	112 (6.7)	70 (8.3)	41 (5.0)	<.001	3.7
Insomnia	70 (58.3)	63 (61.2)	7 (43.8)	591 (35.3)	332 (39.3)	256 (31.2)	<.001	1.6
Attempted suicide	19 (15.8)	17 (16.5)	2 (12.5)	35 (2.1)	32 (2.8)	15 (1.3)	<.001	7.5
Self-injury	19 (15.8)	18 (17.5)	1 (6.3)	47 (2.8)	24 (3.8)	11 (1.8)	<.001	5.6
Eating disorders	32 (26.7)	30 (29.1)	2 (13)	102 (6.1)	71 (8.4)	30 (3.7)	<.001	5.6
Physical health								
Gastrointestinal disease	29 (24.2)	23 (22.3)	6 (37.5)	272 (16.3)	143 (16.9)	127 (15.5)	.03	1.5
Fibromyalgia	18 (15.0)	17 (16.5)	1 (6.3)	62 (3.7)	46 (5.5)	16 (2.0)	<.001	1.5
Musculoskeletal	37 (30.8)	31 (30.1)	5 (31.3)	198 (11.8)	113 (13.4)	84 (10.2)	<.001	2.6
Chronic pain	57 (47.5)	47 (62.5)	6 (37.5)	331 (19.8)	181 (21.4)	148 (18.0)	<.001	2.4

*Note.* SA = sexual assault; PTSD = posttraumatic stress disorder.

a Eight of the respondents in the non-SA group and one in the SA group did not provide their gender.

*p values for differences in proportions between participants with and without SA.

### QoL and Self-Efficacy

The SA group scored significantly lower on QoL (68.1 vs. 75.9, *p* = .04) compared with the non-SA group (Table 3). There was no significant difference between the genders in either group regarding QoL scores. The SA group also scored significantly lower on the GSE rating (26.8 vs. 29.2, *p* < .001) compared with the non-SA group. There were no significant differences between men and women’s GSE scores in the SA group, but there was a significant difference in the GSE scores between the genders in the non-SA group (*p* < .001). Respondents in the SA group who fulfilled the PTSD symptom criteria reported significantly lower levels of GSE than respondents in the SA group who did not have PTSD symptoms: 22.8 (95% CI [25.4, 28.1]) versus 29.2 (95% CI [28.9, 29.5]; *p* = .002). No significant difference was found between genders.

**Table 3.** QoL and GSE Among Participants With and Without Experience of SA.

	Participants With SA			Participants Without SA			*p* *	ES*
	Total	Women	Men	Total	Women	Men		
QoL	68.1	67.1	75.7	75.9	76.2	75.8	.039	.30
95% CI	[63.9, 72.2	[62.7, 71.6]	[64.6, 86.7]	[74.9, 76.9]	[74.8, 77.6]	[74.3, 77.2]		
GSE	26.8	26.8	26.7	29.2	28.6	29.7	<.001	.38
95% CI	[25.4, 28.1	[25.3, 28.3]	[22.4, 30.9]	[28.9, 29.5]	[28.2, 29.1]	[29.3, 30.1]		

*Note.* Higher scores represent higher QoL and higher GSE. Effect sizes (ES) are Cohen’s *d*. QoL = higher quality of life; GSE = general self-efficacy; SA = sexual assault; CI: confidence interval.

*p values and ES related to differences between the total scores for both groups.

## Discussion

### Prevalence of SA

In this study, 10.9% of women and 1.9% of men reported that they had experienced SA during their lifetime. Our finding regarding the prevalence among men is consistent with Breiding and colleagues’ (2014) finding, who reported 1.7%, whereas the prevalence among women was lower than in previous studies (Black et al., 2011; Elliott et al., 2004; Masho et al., 2005). The discrepancies between studies are most likely due to differences in the definitions used by the various researchers (i.e., the cited studies included inappropriate touching the breast, buttocks, or genital area, which were not included here), the approach to screening for SA and the method of data collection.

It seems reasonable to expect a cumulative exposure to assaults with increasing age; however, we found a lower lifetime prevalence of SA in those aged ≥66 years compared with younger age. Explanation for this could be recall bias and differences across age cohorts in attitudes toward violence. SA can occur at any point in life. In this study, SA occurred more frequently when the victims were aged <30 years, regardless of gender, which is consistent with previous research (Elliott et al., 2004; McLean, 2013; Riggs et al., 2000). However, a higher proportion of men than women reported experiencing SA as a child or adolescent. This is in contrast to the findings of a meta-analysis conducted in 2009 that analyzed 22 countries (including Norway), reporting the global experience of childhood and adolescent SA as being highest among female participants (Wihbey, 2011).

### Mental Health

Although an average of 25 years had passed since the SA, men and women who had experienced SA reported significantly more mental health problems than did individuals who had not. Our data are consistent with previous research in this area and suggest that SA is a trauma-inducing event for both men and women and may have long-term effects for many victims. It is commonly believed that men are less negatively affected by SA. However, in the view of Duchesne and co-workers (2018) and our findings, it appears that psychological complaints are similar among male and female victims of SA. To provide help for men, they must also be recognized as victims by help providers and institutions. However, recent studies have found that gender-biased and “ideal victim” perspectives are still prevalent within several relevant institutions (Javaid, 2017). Police officers often view male victims as “unimportant” and “not serious” and generally assume that “men cannot be raped” (Javaid, 2017, p. 16). According to Depraetere and co-workers (2018), social and political changes that challenge prevailing stereotypical perceptions of sexual victims are needed to improve support services for male victims of SA.

It has been reported that as a result of these negative outcomes, many—especially those with symptoms of PTSD—will use depressant-lowering agents such as alcohol, whereas some will injure themselves (Mason & Lodrick, 2013). Here we found no difference regarding alcohol consumption between the SA and non-SA groups. However, we did find that the SA group had a 6 times higher risk for self-injury compared with the non-SA group. In line with previous research, we also found that the SA group had a higher risk for attempting suicide during their lifetime compared with the non-SA group. It has been documented that SA is more strongly associated with suicidality than other forms of trauma and this association appears to be independent of co-occurring disorders (Stein et al., 2010). One reason for suicidality could be shame. The relationship between SA and shame is well documented (Feiring & Taska, 2005; Sable et al., 2006), as is the relation between shame and eating disorders (Goss & Allan, 2009; Troop et al., 2008). Sexual trauma has been identified as one potential pathway to the development and maintenance of eating disorders (Hollande & Satori, 2019; Madowitz et al., 2015). Here we found that the SA group had a 6 times higher risk of eating problems than did the non-SA group.

We found that there was no significant difference between the SA group and the non-SA group regarding employment status. However, our finding that a higher proportion of individuals in the SA group received disability benefits compared with their counterparts suggest that SA might affect some adult survivors’ ability to work. Also, previous research has found that SA and the related trauma response can disrupt survivors’ employment in several ways (Haagsma et al., 2012; Loya, 2015; Tjaden & Thoennes, 2006). The reasons for not being able to work probably arise from numerous negative mental health outcomes found in this and previous studies (Campbell et al., 2009; Saps et al., 2009; Walsh et al., 2012). Reported negative mental health outcomes among victims of SA often include PTSD, anxiety, and depression.

### Physical Health

Research on the long-term effects of SA has primarily focused on mental health outcomes. However, some studies have found associations between SA and somatic outcomes, such as pain, fibromyalgia, and GI disorders (for an overview, see Paras et al., 2009). Here we found that there was approximately a 3 times higher risk of having chronic pain and/or musculoskeletal disease in the SA group compared with the non-SA group. Also, there appears to be increased risk of fibromyalgia and GI disorders among individuals who have experienced SA. Interestingly, there was no significant difference between genders. Most previous research regarding physical health and SA has focused on female victims. Only two studies were identified that investigated the association between GI disorders and SA in both male and female victims. Walker et al. (1993) found that only female victims of SA reported GI disorders, while Bonvanie et al. (2015) found that SA was associated with GI disorders in adolescents, both boys and girls. A meta-analysis found that SA and GI complaints were related, but not fibromyalgia (Paras et al., 2009). However, the studies surveyed only included female victims. Thus, further research is needed to clarify to what extent SA is associated with GI disorders and fibromyalgia in male victims.

### QoL and Self-Efficacy

In view of previous studies and our findings, it appears that having experienced SA during one’s life is associated with lifetime occurrence of multiple health problems for both men and women, which may also reduce a persons’ perceived QoL. A history of SA is associated with numerous negative mental health outcomes, which can affect the survivors’ employment and income-earning capacity (Haagsma et al., 2012; Livingston, 2009), which again may have a negative impact on a person’s perceived QoL. Here we found no difference in employment rates between the SA group and non-SA group. However, we found that a significantly higher percentage of the SA group received disability benefits. This might also be why the SA group perceived their QoL to be lower than the non-SA group.

The GSE rating has been linked with various physical and mental health outcomes (Lerdal et al., 2017; Nygaard et al., 2017). In view of the higher prevalence of both mental and physical health problems in the SA group in our study, it is not surprising that they reported lower self-efficacy and poorer QoL than the non-SA group. The experience of SA could affect a persons’ view of themselves, others, and the world, as well as resulting in low self-esteem and self-blaming, which results in lower levels of self-efficacy (Brunes & Heir, 2018). According to Diehl and Prout (2002), self-efficacy is a key psychological component for restoring functioning and health after experiencing trauma. Here, lower perceived self-efficacy was associated with higher rates of PTSD symptoms in both male and female victims of SA. Thus, the study confirms the findings of Voller and co-workers (2015), showing that lower perceived self-efficacy is also associated with more severe psychiatric symptoms in male victims.

### Strengths and Limitations of the Study

This study used a nationally representative sample, including both female and male participants. The anonymous survey approach appeared to be well suited for obtaining reliable self-reports about the sensitive topics investigated in this study. The small number of men reporting SA limited our ability to perform statistical tests of gender differences while adjusting for other variables. An overall 36% response rate raises the question of whether the sample was representative of its target population. However, lower response rates do not necessarily produce more nonresponse errors (Holbrook et al., 2008). A prior study based upon the same sample demonstrated that the sample characteristics and estimates are consistent with population data from Statistics Norway (Schou-Bredal et al., 2017). Thus, we consider our sample to be representative of the general Norwegian population. We used standardized, validated measurements to assess lifetime SA and PTSD symptoms.

Most previous studies have been limited because the composition of SA groups has been largely restricted to those having experienced SA in childhood or adolescence, and in female or adult victims (Dworkin et al., 2017). By contrast, this study included lifetime SA in both genders. Furthermore, the use of anonymous questionnaires might increase the reliability of self-reporting about sensitive topics such as SA. One potential limitation of this study is recall bias, as SA in childhood might very likely be underreported (Paras et al., 2009) and thus influence the associations between SA and subsequent mental and physical health.

## Conclusion

To our knowledge, this is the first study to investigate self-reported lifetime SA in men and women in the Norwegian general population, and also the first to compare these victims with non-victims with regard to GSE, QoL, and a broad range of health conditions. The study found that the prevalence of SA in the Norwegian general population was 6.7%, and considerably higher among women (10.9%) than among men (1.9%). Having experienced SA during one’s life was associated with lifetime occurrence of multiple health problems for both genders. The health differences included, but were not restricted to, mental health problems. Prevalence rates for all of the included physical health conditions were higher for those who had experienced SA, with a risk of illness between 1.5 and 2.6 times higher compared with their counterparts without SA experience. For the listed mental health problems, by contrast, the risk of illness was between 1.6 (insomnia) and 7.5 (attempted suicide) times higher than for those without lifetime SA. Persons with lifetime SA also reported poorer QoL and lower GSE. Moreover, they were less likely to be married or cohabitating and more likely to receive disability benefits. In view of the results, it seems clear that SA is strongly related to population diversity in terms of health, coping resources, and work participation. We conclude that SA experience is a burden that increases the risk of experiencing a wide range of other burdens in life.
